# Exploratory longitudinal cohort study of modest bilirubin-driven biochemical liver alterations after SARS-CoV-2 infection in a selected subgroup of patients with Wilson’s disease and liver cirrhosis

**DOI:** 10.1186/s12876-026-04840-3

**Published:** 2026-04-20

**Authors:** Sebastian Köhrer, Maximilian Lukas Brand, Viola Leidner, Holger Zimmer, Andrea Langel, Jessica Langel, Patrick Michl, Isabelle Mohr

**Affiliations:** 1 Internal Medicine IV, Department of Gastroenterology, University Hospital Heidelberg, Heidelberg, Germany; 2 Internal Medicine I, Department of Endocrinology, Diabetology, Metabolic Diseases and Clinical Chemistry, University Hospital Heidelberg, Heidelberg, Germany

**Keywords:** Wilson’s disease, copper metabolism, SARS-CoV-2 infection, COVID-19, Long-COVID, Liver cirrhosis

## Abstract

**Background:**

Wilson’s Disease (WD) is a rare genetic disorder characterized by impaired hepatic copper elimination, potentially leading to chronic liver disease and cirrhosis. Although SARS-CoV-2 primarily affects the respiratory tract, hepatic involvement has been described. Using WD as a well-characterized model of a rare chronic liver disease with standardized long-term follow-up, we exploratively assessed short- and intermediate-term associations between SARS-CoV-2 infection and liver function as well as copper metabolism in patients with and without cirrhosis.

**Methods:**

In this longitudinal real-world study, we analyzed 71 WD patients (49 without cirrhosis, 22 with cirrhosis) with confirmed SARS-CoV-2 infection. Laboratory parameters, 24-hour urinary copper excretion (24-UCE), and transient elastography were assessed before infection (t0), shortly after infection (t1), and >12 months post-infection (t2). Effect sizes (r) were calculated for intraindividual comparisons. Clinical severity, vaccination status, and long-COVID symptoms were recorded.

**Results:**

No patient required hospitalization. In WD patients without cirrhosis, liver enzymes, cholestasis markers, and copper metabolism remained stable across all timepoints, with predominantly very small effect sizes (|r| < 0.3), indicating no clinically meaningful changes. In contrast, cirrhotic WD patients exhibited moderate to large short-term effects in transaminases and GGT (r up to 0.85) following infection. Bilirubin showed the most pronounced and persistent alteration in cirrhotic patients, with a sustained but only moderate effect (r ≈ 0.6) at long-term follow-up. Partial biochemical recovery was observed, yet normalization remained incomplete. Liver stiffness and copper parameters did not change significantly in either group.

**Conclusions:**

SARS-CoV-2 infection was associated with persistent biochemical alterations, particularly elevated bilirubin levels of moderate magnitude in WD patients with cirrhosis, while global liver function parameters remained largely stable. These findings highlight the need for close post-infection monitoring of WD patients with cirrhosis, even in mild SARS-CoV-2 infections, to mitigate potential hepatic complications and guide ongoing management in this vulnerable population. Nevertheless, these observations are hypothesis-generating and should be interpreted cautiously due to the small, selected cirrhotic subgroup, the lack of appropriate control groups, and the delayed timing of post-infection assessments.

**Supplementary Information:**

The online version contains supplementary material available at 10.1186/s12876-026-04840-3.

## Background

Wilson’s Disease (WD) is an autosomal recessive disorder caused by mutations in the ATP7B gene, leading to impaired hepatic copper elimination and systemic copper accumulation, primarily affecting the liver and brain [1, 2]. Clinical manifestations are heterogeneous: nearly all patients present with chronic liver disease, ranging from mild transaminase elevations to cirrhosis with impaired hepatic function [3, 4]. Neurological symptoms, occurring in 40–60% of patients, typically arise years after initial liver involvement, most often in the second or third decade of life [5]. They range from mild tremor or dysarthria to severe ataxia or chorea. Some patients also experience psychiatric symptoms or involvement of other organs [6]. In the present study, we therefore use WD as a model of a rare, well-characterized chronic liver disease with long-term follow-up to descriptively explore intraindividual biochemical changes before and after SARS-CoV-2 infection, without aiming to demonstrate a WD-specific mechanistic effect. This makes WD particularly suitable for studying the hepatic consequences of systemic infections such as COVID-19 under controlled clinical conditions. 

SARS-CoV-2, the virus causing COVID-19, emerged in 2019 and caused a global pandemic with profound health, economic, and societal impacts. While primarily a respiratory illness, systemic involvement is frequent, including hepatic injury [7]. Clinical severity varies from asymptomatic to critical illness, with 20–40% of infections remaining asymptomatic [8, 9]. Vaccination and post-infection immunity have shifted the majority of cases toward milder disease; however, SARS-CoV-2 continues to pose a substantial burden, with hospitalization rates in certain risk groups exceeding historical influenza-related rates [10]. Patients with chronic liver disease (CLD), particularly those with cirrhosis, are at increased risk of severe COVID-19 outcomes. In the international APCOLIS study, 20% of cirrhotic patients developed acute decompensation or acute-on-chronic liver failure, and a Child–Pugh score ≥ 9 strongly predicted mortality [11]. Similar findings from multicenter cohorts and systematic reviews confirm that CLD is associated with higher hospitalization rates, higher chance of admission to an intensive care unit, and increased mortality compared with non-CLD patients [12, 13]. Reported mortality in cirrhotic patients ranged from 16.6% to 51.3%, depending on population and pre-vaccination status [14]. Predictors of poor outcome include elevated bilirubin, prolonged INR, and requirement for respiratory support [15]. Mechanisms underlying these complications involve direct viral injury to hepatocytes and cholangiocytes via ACE2 receptor expression, immune-mediated injury, cytokine storm, hypoxic and ischemic hepatopathy, drug-induced liver injury, and hepatitis B reactivation under immunosuppressive therapy [16–19]. Clinical practice recommendations emphasize close monitoring of liver function, INR, and bilirubin, early identification of decompensation, vaccination, management of comorbidities, and prophylaxis for hepatitis B where appropriate [17, 20]. COVID-19 can present with mild respiratory symptoms or, in severe cases, pneumonia and multiorgan involvement, often accompanied by elevated transaminases, LDH, inflammatory markers, and coagulation abnormalities [21, 22]. Recent studies suggest that patients with WD are particularly susceptible to long-COVID, experiencing persistent mental and physical health impairments after SARS-CoV-2 infection [23]. However, the long-term effects of COVID-19 on liver function and copper metabolism in WD remain poorly understood. Understanding these consequences is crucial for infection prevention, monitoring, and management in this vulnerable population. To date, short- and long-term effects of SARS-CoV‐2‐infection on liver function in patients with WD remain unclear. Our study aims to examine these effects using WD as a model for hepatic and neuropsychological chronic disease. Because first post-infection assessments were performed during routine outpatient follow-up approximately three months after SARS-CoV-2 infection, our analysis does not capture the acute phase of COVID-19 but rather short- and long-term post-infectious liver biochemistry.

## Methods

**Fig. 1:**
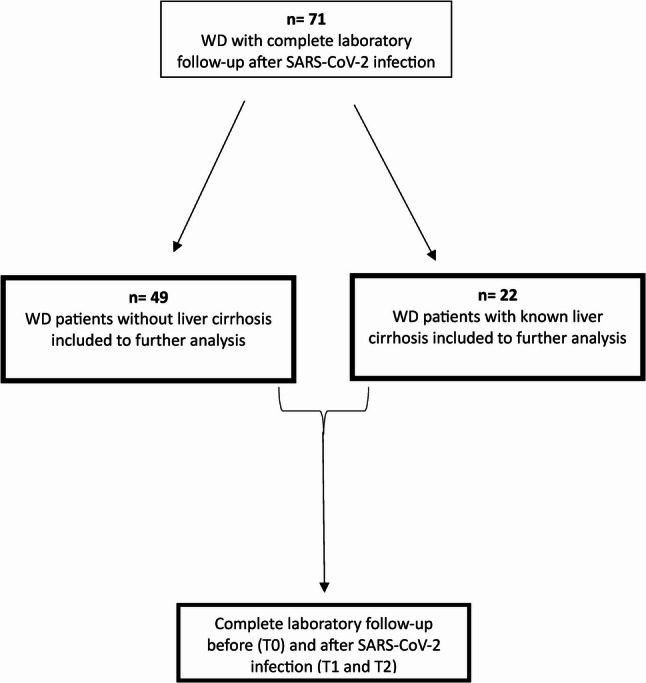
Study design. This study screened all 234 patients with confirmed Wilson Disease. Of these, 192 patients (82%) had a documented SARS-CoV-2 infection and were therefore eligible for inclusion. Among the 192 eligible patients, 71 had complete follow-up data, including laboratory results, abdominal ultrasound imaging, and non-invasive fibrosis assessment, and were included in the final analysis

This study screened all 234 patients with confirmed Wilson’s Disease (WD; Leipzig score ≥ 4) who attended our outpatient clinic between January 2020 and December 2023. Of these, 192 patients (82%) had a documented SARS-CoV-2 infection and were therefore eligible for inclusion. Exact virus variants were not routinely documented due to clinical standard-of-care. Based on infection timing, most infections likely occurred during the Omicron-dominant period. Clinical and laboratory data were analyzed at three predefined timepoints: t0, defined as the last available outpatient visit prior to SARS-CoV-2 infection; t1, defined as the first outpatient visit after infection; and t2, defined as the most recent available outpatient visit following infection. Clinical data were collected accordingly at each timepoint. Of 192 eligible patients, 71 had complete follow-up data (laboratory results, abdominal ultrasound imaging, and non-invasive fibrosis assessment) and were included. 121 patients were excluded due to incomplete longitudinal laboratory (explicitly missing biochemical values at t2) and imaging follow-up, as complete datasets before and after SARS-CoV-2 infection were required for reliable within-patient comparisons. Supplemental Table 1 presents baseline characteristics of excluded patients. The patient inclusion process is summarized in Fig. 1.

Included patients were stratified into two groups: WD patients without liver cirrhosis (LC) and WD patients with confirmed LC. LC was defined by at least one of the following criteria: histological evidence of cirrhosis on liver biopsy; clinical signs of portal hypertension, including ascites, esophageal varices with or without prior variceal bleeding, or significant splenomegaly accompanied by thrombocytopenia; or F4 fibrosis on transient elastography. For both groups, laboratory parameters, abdominal ultrasound findings, and non-invasive liver stiffness measurements were analyzed. Available laboratory test results were analyzed to assess any change in liver function before and after an infection with SARS-CoV-2. Considered values were transaminases (aspartate-aminotransferase (AST/GOT) and alanine-aminotransferase (ALT/GPT), respectively), parameters of cholestasis (alkaline phosphatase (AP), gamma-glutamyltransferase (GGT)), as well as parameters of liver excretion (serum bilirubin) and synthesis capacity (international normalized ratio (INR), choline esterase (ChE)). In addition, detailed clinical data were collected, including WD-specific treatment and treatment duration, parameters of copper metabolism, number of confirmed SARS-CoV-2 infections, SARS-CoV-2 vaccination status, and the presence of long-COVID symptoms. Patients were asked to retrospectively assess symptom severity before and after their SARS-CoV-2 infection using a long-COVID specific, non-standardized but clearly structured questionnaire (Supplemental Table 2). The questionnaire explicitly distinguishes between new symptoms and worsening of existing symptoms to improve recall accuracy. Long-COVID was defined as the occurrence of new symptoms or worsening of pre-existing symptoms—such as dyspnea, chest pain, fatigue, cognitive impairment (“brain fog”), reduced exercise capacity, or concentration difficulties—of at least moderate intensity that persisted until completion of the patient survey. Pre-existing symptoms and their baseline severity were cross-checked with available patient records; symptoms documented prior to infection without evidence of deterioration were not classified as Long-COVID. Given the retrospective design, the use of a non-validated questionnaire, and the potential overlap between Long-COVID symptoms and pre-existing WD-related neurological or psychiatric manifestations, these data were considered exploratory and were not used to derive prevalence estimates. Additionally, non-invasive liver stiffness measurements were performed using transient elastography (FibroScan^®^, Echosens, Paris, France). Liver stiffness and the Controlled Attenuation Parameter (CAP) were recorded as outcome measures.

### Statistical analysis

All data were pseudonymized and collected in an Excel database before being exported to IBM SPSS Statistics (version 27.0; IBM, Chicago, IL, USA) for analysis. Descriptive statistics included frequencies, measures of central tendency (mean and median), and measures of dispersion. Prior to analysis, assumptions required for parametric repeated-measures approaches were evaluated and found to be violated (including sphericity, normality, and homogeneity of variances). Given these violations and the limited sample size, repeated-measures ANOVA and mixed-effects models were considered inappropriate. Multivariable modeling was not performed due to the limited sample size relative to the number of potential confounders, which would have resulted in model overfitting. Comparisons between timepoints were therefore performed using the Wilcoxon signed-rank test as a non-parametric approach. Effect sizes (r-value) were calculated and corresponding 95% confidence intervals added. Effect sizes were calculated to complement p-values and to help distinguish statistically from clinically relevant changes; however, large r-values in the context of very small absolute differences or minimal variance were not interpreted as clinically meaningful.

## Results

### Study cohort

Baseline characteristics of patients with Wilson’s Disease (WD) without liver cirrhosis (non-LC) with liver cirrhosis (LC) are summarized in Table 1 (non-LC) and 2 (LC).

**Table 1:** Patient baseline characteristics for WD patients without liver cirrhosis

Category	Parameter	Value
Study population	Patients included, n Male, % Female, %	49 40.8% 59.2%
Demographics	Age at study inclusion, years Age at diagnosis, years	37 (range 19 to 70; SD 15.2) 16 (range 1 to 50; SD 10.9)
Clinical phenotype of WD	Primarily hepatic, n (%) Primarily neurological, n (%) Mixed phenotype, n (%)	34 (69.4) 6 (12.2) 9 (18.4)
Treatment at study inclusion	D-penicillamine, n (%) Trientine, n (%) Zinc, n (%) Combination therapy (Zinc + Trientine), n (%)	15 (20.6) 33 (67.3) 3 (6.1) 2 (4.1)
Disease characteristics	Overall treatment duration, years	24 (range 3 to 56; SD 15.7)
SARS-CoV-2 infection	> 1 infection, n (%) two infections, n (%) Hospitalization due to SARS-CoV-2, n (%)	49 (100) 6 (12.0) none
Long-COVID Symptoms	After 1st infection, n (%) After 2nd infection, n (%)	4 (28.6) 14 (28.6) 2 (4.1)
Vaccination status	First vaccination, n (%) Second vaccination, n (%) Third vaccination, n (%)	47 (95.9) 47 (95.9) 42 (85.7)
Timing of laboratory assessment	T0 (pre-infection), months T1 (after-infection), months T2 (after-infection), months	2.7 (2–12; SD 2.9) 3.2 (2.5–3.5, SD 0.2) 16.5 (4–30; SD 5.3)

Table 1 depicts patient baseline characteristics (demographics, treatment, SARS-CoV-2 history, Long-COVID, vaccination status) for 49 WD patients without liver cirrhosis

**Table 2:** Patient baseline characteristics for WD patients with liver cirrhosis

Category	Parameter	Value
Study population	Patients included, n Male, % Female, %	22 63.6% 36.4%
Demographics	Age at study inclusion, years Age at diagnosis, years	46 (range 19 to 73; SD 14.3) 21 (range 11 to 58; SD 10.6)
Child-Pugh Score	Stage A, n (%) Stage B, n (%) Stage C, n (%)	18 (81.8) 4 (18.2) none
Clinical phenotype of WD	Primarily hepatic, n (%) Primarily neurological, n (%)	19 (86.4) 3 (13.6)
Treatment at study inclusion	D-penicillamine, n (%) Trientine, n (%)	10 (45.5) 12 (54.5)
Disease characteristics	Overall treatment duration, years	23 (range 2 to 56; SD 15.3)
SARS-CoV-2 infection	> 1 infection, n (%) two infections, n (%) Hospitalization due to SARS-CoV-2, n (%)	22 (100) 2 (9.1) none
Long-COVID Symptoms	After 1st infection, n (%) After 2nd infection, n (%)	2 (9.1) 2 (9.1)
Vaccination status	First vaccination, n (%) Second vaccination, n (%) Third vaccination, n (%)	17 (77.3) 17 (77.3) 14 (68.2)
Timing of laboratory assessment	T0 (pre-infection), months T1 (after-infection), months T2 (after-infection), months	3.5 (2–8; SD 2.2) 2.8 (2.4–3.2, SD 0.1) 16.2 (7–32; SD 6.9)

Table 2 summarizes patient baseline characteristics for 22 WD patients with liver cirrhosis including baseline demographics, disease characteristics, treatment status, SARS-CoV-2 infection history, Long-COVID incidence, and vaccination uptake

To assess potential selection bias, we compared available baseline characteristics between included (*n* = 71) and excluded patients (*n* = 121; see Supplemental Table 1). Baseline parameters did not differ substantially between groups: excluded patients showed a median age of 38 years, a median age at diagnosis of 18 years, and a prevalence of liver cirrhosis of 25%, which was comparable to the analyzed cohort. Disease phenotypes and WD-specific therapeutic regimens were comparable either.

A total of 71 WD patients with complete follow-up data were included in the analysis. Of these, 49 patients had no history of liver cirrhosis, while 22 patients had confirmed cirrhosis. In the non-LC group, 20 patients (40.8%) were male and 29 (59.2%) were female. The median age at inclusion was 37 years (range 19–70; SD 15.2 years). WD presentation was predominantly hepatic in 34 patients (69.4%), primarily neurological in 6 patients (12.2%), and mixed hepatic–neurological in 9 patients (18.4%). Regarding treatment, 15 patients (30.6%) received D-penicillamine, 33 patients (67.3%) trientine, and 3 patients (6.1%) zinc, of whom two also received trientine. Median treatment duration was 24 years (range 3–56; SD 15.7 years). In the LC group, 14 patients (63.6%) were male and 8 (36.4%) were female. Eighteen patients had Child–Pugh A cirrhosis and four had Child–Pugh B cirrhosis; no patient had Child–Pugh C disease. Median age at inclusion was 46 years (range 19–73; SD 14.3 years). WD presentation was primarily hepatic in 19 patients (86.4%) and mixed in 3 patients (13.6%); no patient exhibited a purely neurological phenotype. Ten patients (45.5%) were treated with D-penicillamine and twelve (54.5%) with trientine; none received zinc. Median treatment duration was 23 years (range 2–56; SD 15.3 years). For timepoint t0, the last routine blood sample prior to confirmed SARS-CoV-2 infection was used. The mean interval between t0 and infection was 2.7 months (range 2–12; SD 2.9 months) in the non-LC group and 3.5 months (range 2–8; SD 2.2 months) in the LC group. Timepoint t1 represented the first available blood sample after infection. The mean interval from t0 to t1 was 3.2 months (2.5–3.5; SD 0.2 months) in WD patients without LC and 2.8 months (2.4–3.2; SD 0.1 months) in WD patients with LC after SARS-CoV-2 infection. T2 corresponded to the last available follow-up sample. Mean follow-up after infection was 16.5 months (range 4–30; SD 5.3 months) in the non-LC group and 16.2 months (range 7–32; SD 6.9 months) in the LC group.

### SARS-CoV-2 baseline characteristics

None of the patients required hospitalization for acute COVID-19 or related complications. Two consecutive SARS-CoV-2 infections were documented in 6 of 49 non-LC patients (12%) and in 2 of 22 LC patients (9.1%); no patient experienced more than two infections. Vaccination coverage was high: 47 non-LC patients (95.9%) received at least two vaccinations and 42 (85.7%) received a third dose. Vaccination status included all vaccinations received up to the noted infection. In the LC group, 17 patients (77.3%) were vaccinated at least twice and 14 (68.2%) received a third dose. Long-COVID, defined as new or worsening symptoms such as dyspnea, chest pain, fatigue, cognitive impairment, reduced exercise capacity, or concentration difficulties, was reported by 14 non-LC patients (28.6%) and 2 LC patients (9.1%). Median Long-COVID symptom duration was 10.9 months (range 2.5 to 40.8; SD 6.0 months). The patient questionnaire and its detailed results within the cohort are presented in Supplemental Table 2.

### Laboratory results

In patients with WD but no LC, neither transaminases nor cholestasis parameters were significantly affected both shortly after SARS-CoV-2 infection (t1) or in the long term (t2). Overall, the analyses demonstrated almost exclusively very small effect sizes (|r| < 0.3), indicating minimal quantitative changes over time. Although some comparisons reached statistical significance based on p-values, these findings did not translate into clinically meaningful alterations considering the respective effect sizes. The overall course remained clinically stable across all assessed time points. Although the effect size for INR appeared numerically large, this was driven by minimal variance and very small absolute differences; coagulation status remained clinically stable, and we did not interpret this as a meaningful change.

**Table 3:** Overall descriptive statistics for key laboratory results before, during and after SARS-CoV-2 infection for all patients without liver cirrhosis

Parameter	Last blood sample before SARS-CoV-2 infection (T0) *		First blood sample after SARS-CoV-2 infection (T1)		Last blood sample after SARS-CoV-2 infection (T2)		T0 vs. T1	T1 vs. T2	T0 vs. T2
	*n*	Mean (95% CI)	*n*	Mean (95% CI)	*n*	Mean (95% CI)	*r*-value	*r*-value	*r*- value
AST/GOT [U/l]	49	32 (28.4–35.6)	49	36 (27.4–44.6)	49	31 (25.7–36.3)	+ 0.32	−0.17	−0.08
ALT/GPT [U/l]	49	38 (28.6–47.4)	49	37 (24.4–49.6)	49	42 (31.6–52.4)	−0.03	+ 0.11	+ 0.12
GGT [U/l]	49	29 (23.7–34.3)	49	28 (19.0–37.0)	49	29 (23.4–34.6)	−0.05	+ 0.03	0.00
AP [U/l]	49	90 (78.5–101.5)	49	92 (82.2–101.8)	49	87 (78.3–95.7)	+ 0.05	−0.15	−0.08
Bilirubin [mg/dl]	49	0.8 (0.71–0.89)	49	0.8 (0.68–0.92)	49	0.7 (0.61–0.79)	0.00	−0.25	−0.33
INR	49	1.0 (0.97–1.03)	49	1.1 (1.07–1.13)	49	1.0 (0.97–1.03)	+ 1.00*	−1.00*	0.00
Cholinesterase [kU/l]	49	7.6 (6.9–8.3)	49	7.9 (7.0–8.8)	49	8.3 (7.5–9.1)	+ 0.13	+ 0.13	+ 0.29
WBC [/nl]	49	5.8 (5.3–6.3)	49	5.6 (5.0–6.2)	49	5.4 (5.0–5.8)	−0.12	−0.11	−0.24
Thrombocytes [/nl]	49	226 (199.4–252.6)	49	220 (195.8–244.2)	49	3.5 (− 18.7–25.7)	−0.07	+ 0.18	+ 0.10
24 h Urinary copper excretion [µmol/d]	49	2.4 (1.94–2.86)	49	3.0 (2.04–3.96)	49	2.5 (1.63–3.37)	+ 0.38	−0.15	+ 0.06
Serum copper [µmol/l]	49	4.1 (2.8–5.4)	49	4.2 (2.9–5.5)	49	4.3 (3.2–5.4)	+ 0.02	+ 0.02	+ 0.04
BMI [kg/m^2^]	49	23.7 (22.5–24.9)	49	24.3 (23.1–25.5)	49	24.1 (22.9–25.3)	+ 0.15	−0.05	+ 0.10
Stiffness [kPa]	49	6.7 (5.7–7.7)	49	6.4 (5.5–7.3)	49	6.2 (5.4–7.0)	−0.08	−0.06	−0.14
CAP [dB/m]	49	243.3 (226.9–259.7)	49	243.6 (227.6–259.6)	49	243.5 (228.9–258.1)	+ 0.01	−0.00	+ 0.00

Table 3 summarizes the laboratory findings in patients with WD and no LC at the different timepoints before and after SARS-CoV-2 infection, including key laboratory parameters of liver function and copper metabolism as well as BMI and Fibroscan.

In patients with WD and LC, moderate to large short-term effects were observed for several liver parameters, including AST, ALT, GGT, and bilirubin. Among these markers, bilirubin showed a persistent effect over time, with an effect size of approximately *r* ≈ 0.6. This led to a progression of the Child-Pugh Score from stage A to stage B in three out of 22 patients (13.6%). Increasing bilirubin levels led to exceeding the clinically relevant threshold of total bilirubin >2x of upper limit of normal in four cases (18.2%). No evidence of hepatic decompensation was reported within the study timeline. Although many parameters demonstrated partial reversal from t1 to t2, the recovery was not complete, suggesting residual alterations beyond the short-term phase.

**Table 4:** Overall descriptive statistics for key laboratory results before, during and after COVID-19 infection for all patients with liver cirrhosis

Parameter	Last blood sample before SARS-CoV-2 infection (T0)		First blood sample after SARS-CoV-2 infection (T1)		Last blood sample after SARS-CoV-2 infection (T2)		T0 vs. T1	T1 vs. T2	T0 vs. T2
	*n*	Mean (95% CI)	*n*	Mean (95% CI)	*n*	Mean (95% CI)	*r*-value	*r*- value	*r*- value
AST/GOT [U/l]	22	31 (26.6–35.4)	22	38 (30.2–45.8)	22	33 (26.4–39.6)	**+ 0.70**	−0.28	+ 0.20
ALT/GPT [U/l]	22	32 (24.9–39.1)	22	42 (30.1–53.9)	22	36 (26.4–45.6)	**+ 0.62**	−0.22	+ 0.25
GGT [U/l]	22	38 (9.6–66.4)	22	93 (36.1–149.9)	22	42.5 (− 13.3–98.3)	**+ 0.85**	−0.39	+ 0.07
AP [U/l]	22	96 (81.7–110.3)	22	102 (83.4–120.6)	22	86.5 (71.8–101.2)	+ 0.18	−0.37	−0.29
Bilirubin [mg/dl]	22	0.7 (0.48–0.92)	22	1.3 (0.86–1.74)	22	1.0 (0.25–1.75)	**+ 1.20**	−0.30	**+ 0.60**
INR	22	1.14 (1.10–1.18)	22	1.16 (1.07–1.25)	22	1.11 (1.02–1.20)	+ 0.20	−0.25	−0.30
Cholinesterase [kU/l]	22	6.9 (5.8–8.0)	22	6.3 (4.9–7.7)	22	7.2 (5.8–8.6)	−0.24	+ 0.29	+ 0.12
Albumin [g/l]	22	43.6 (41.8–45.4)	22	41.2 (39.0–43.4)	22	44.2 (42.0–46.4)	−0.57	+ 0.61	+ 0.14
WBC [/nl]	22	4.0 (3.6–4.4)	22	4.1 (3.6–4.6)	22	4.3 (3.9–4.7)	+ 0.10	+ 0.18	+ 0.30
Thrombocytes [/nl]	22	129 (105.4–152.6)	22	134 (108.9–159.1)	22	118 (89.6–146.4)	+ 0.09	−0.28	−0.21
24 h Urinary copper excretion [µmol/d]	22	2.4 (1.6–3.2)	22	2.7 (1.4–4.0)	22	2.2 (0.2–4.2)	+ 0.17	−0.17	−0.11
Serum copper [µmol/l]	22	6.1 (4.6–7.6)	22	5.2 (4.2–6.2)	22	6.4 (4.6–8.2)	−0.26	+ 0.55	+ 0.09
BMI [kg/m^2^]	22	24.9 (22.8–27.0)	22	24.5 (22.6–26.4)	22	24.9 (22.9–26.9)	−0.08	+ 0.09	0.00
Stiffness [kPa]	22	13.6 (11.2–16.0)	22	13.0 (9.2–16.8)	22	13.3 (8.6–18.0)	−0.11	+ 0.03	−0.06
CAP [dB/m]	22	220 (189.5–250.5)	22	223 (202.9–243.1)	22	211 (188.7–233.3)	+ 0.04	−0.26	−0.13

Table 4 summarizes the laboratory findings in patients with WD and LC at timepoints before and after SARS-CoV-2 infection, including key laboratory parameters of liver function and copper metabolism as well as BMI and Fibroscan

### Copper status before and after SARS-CoV-2 infection

In both the non- and the LC-group, serum copper and 24 h-urinary copper excretion were measured before and after SARS-CoV-2 infection. In both groups, there was no significant change in both serum copper and urinary copper excretion. Mean values and effect sizes for 24 h-urinary copper excretion and serum copper levels revealed no clinically relevant changes throughout the observational period in both groups. Tables 3 and 4 summarize laboratory results for both liver function and copper status in the groups of WD patients without LC (Table 3) and with LC (Table 4).

### Transient elastography before and after SARS-CoV-2 infection

In both groups, liver stiffness and hepatic steatosis were measured using transient elastography (FibroScan^®^) at all three timepoints. Mean values and effect sizes for liver stiffness and hepatic steatosis remained stable without clinically relevant changes throughout the observational period in both groups (Tables 3 and 4). Both groups also showed no clinically relevant change in their respective median body mass index (BMI) measures over the course of the study (Tables 3 and 4, respectively).

## Discussion

Overall, our study provides descriptive, hypothesis-generating evidence of modest, persistent changes in bilirubin in a small, clinically stable cirrhotic WD subgroup after SARS-CoV-2 infection, without demonstrating a WD-specific mechanism or robust causal relationship. We analyzed short- and long-term effects of SARS-CoV-2 infection in patients with Wilson disease (WD). The absence of a non-infected control group limits causal interpretation. Therefore, analyses relied on intraindividual longitudinal comparisons, which reduce interindividual variability but allow only associative conclusions. These comparisons only permit associative conclusions and are hypothesis-generating rather than definitive. The reduced sample size of 71 patients with complete laboratory follow-up data is attributable to the real-world characteristic of this study in a rare liver disease. To assess the potential selection bias due to the exclusion of 121 patients, we compared available baseline characteristics between included and excluded patients. Although baseline demographics, disease phenotype, Wilson’s disease-specific therapy regimens and cirrhosis prevalence were comparable between included and excluded patients, the high proportion of individuals with incomplete longitudinal data may still introduce selection bias, as patients with either particularly stable disease or more severe courses could have been less likely to attend regular follow-up visits. This potential loss-to-follow-up bias may limit the generalizability of our findings and should be considered when interpreting the results. Moreover, this approach resulted in a highly selected subgroup with complete longitudinal data, and selection bias cannot be excluded. The absence of a non-infected WD comparator and of a non-WD cirrhotic control group precludes robust causal inference and limits external validity of the findings.

Due to early diagnosis of WD, lifelong treatment, and regular monitoring, confounding factors such as untreated disease progression or inconsistent follow-up are minimized. Therefore, observations in WD patients may offer valuable insights into infection-related liver dysfunction in other chronic liver diseases. The study cohort reflects a typical WD collective with hepatic and neurological WD manifestations, with slightly fewer neurologically affected patients than described in recent literature [2]. All patients with WD were considered as suffering from chronic liver disease, while those who additionally developed liver cirrhosis (LC) were analyzed separately.

Although mainly affecting the lung, it has been shown that COVID-19 causes symptoms and disturbances in a variety of organs, including the liver. Viral entry of SARS-CoV-2 into the tissues is possible via the angiotensin-converting enzyme 2 receptor [24]. In the liver, this receptor is located on the outside of hepatocytes and, more frequently, cholangiocytes [25]. Previous studies reported markedly increased hospitalization and mortality rates in patients with chronic liver disease, particularly liver cirrhosis, during early phases of the pandemic [17, 26]. A higher Child-Pugh stage cirrhosis was associated with even more pronounced mortality rates [27]. In our cohort, however, hospitalization rate in both analyzed cohorts were 0%, and even patients with greatly impaired liver function (i.e., Child-Pugh stage B cirrhosis) were not suffering from severe SARS-CoV-2 infection. This might reflect several differences in our cohort as opposed to the earlier cohorts. Given the very small, clinically mild cirrhotic subgroup, these findings should be regarded as an exploratory signal of uncertain clinical relevance rather than evidence of a generalized long-term deterioration in liver function.

The absence of severe COVID-19 courses in our cohort likely reflects high vaccination rates [12, 28, 29] and the lack of both Child–Pugh C cirrhosis and hepatic decompensation [13, 15, 30]. Infections occurring predominantly during the Omicron variant-dominant period might also be associated with reduced disease severity [13, 30, 31]. However, exact virus variants were not routinely documented due to our clinical practice at that time, which should be acknowledged as a potential limitation of the study. Based on infection timing, most infections likely occurred during the Omicron-dominant period, which fits to the clinical outcome of non-severe infections without need of hospitalization. While Omicron and its sublineages are characterized by higher transmissibility and immune evasion capability, observational and animal study data suggest a dramatically decreased rate of severe illness and mortality than observed with variants pre-Omicron [30, 31]. It is rather likely that at a larger quantity of patients in this study had at least one infection with the Omicron variant. Additionally, long-COVID symptoms were reported in 28.6% of patients without liver cirrhosis and in 9.1% with liver cirrhosis, with a median symptom duration of 10.9 months. Long-COVID symptom assessment was based on a structured patient survey allowing intraindividual comparison of symptom severity before and after infection, and included standardized severity grading. Pre-existing symptoms were cross-checked with clinical records to improve classification accuracy. However, the assessment Long-COVID symptoms relied on a retrospective, non-validated patient questionnaire, which is prone to recall bias despite cross-checking with records. Although the differentiation between long-COVID and WD-specific neurological symptoms may pose a potential limitation, this challenge was carefully and systematically addressed in the study design. Owing to these methodological limitations, the Long-COVID findings should be interpreted with caution and primarily serve as a hypothesis-generating add-on rather than a central outcome of this study. Nonetheless, future studies should employ prospectively collected, validated Long-COVID instruments to more accurately characterize symptom burden in this specific population. 

In SARS-Cov-2-related acute hepatitis, transient elevation of transaminases has been described regularly, with transaminase levels usually not exceeding 5x the upper limit of normal (ULN) [32, 33]. Transaminase elevation often showed the pattern of greater AST (GOT) than ALT (GPT) elevation, associated with more severe disease [25]. In our cohort, none of the patients showed elevation of transaminases above 5x ULN. Fittingly, liver function parameters in patients with WD following SARS-CoV-2 infection showed small effect sizes across three time points, indicating a largely stable clinical course. Although some statistically significant differences were detected, the magnitude of change was minimal (|r| < 0.3) and did not translate into clinically meaningful deterioration. Even the seemingly large effect size observed for INR was attributable to an extremely small standard deviation rather than to a relevant absolute change, underscoring the importance of interpreting effect sizes in their statistical context. In contrast, moderate to large short-term effects were observed for AST, ALT, GGT, and bilirubin in WD patients with LC, suggesting a transient hepatic impact during the initial phase of infection. Notably, bilirubin demonstrated a persistent moderate effect (*r* ≈ 0.6). While several parameters showed partial normalization from t1 to t2, recovery remained incomplete, indicating residual biochemical alterations beyond the immediate infection period. Taken together, these findings suggest that SARS-CoV-2 infection may be associated with temporary hepatic stress in patients with WD, particularly reflected by sustained bilirubin changes, but does not appear to result in clinically significant deterioration of overall liver function in most cases. Moreover, chronic cholangiopathy, similar to secondary sclerosing cholangitis, has been characterized as a long-term complication of severe COVID-19 [34]. Faruqui et al. reported severe cholestatic disease with biliary tract abnormalities. In their study, only 12 out of over 2000 analyzed patients presented with this syndrome, 5 of which were eventually evaluated for liver transplantation [34]. However, in a study by Hartl et al., about 20% of patients developed progressive cholestasis after SARS-CoV-2 infection with higher risks for patients with metabolic risk factors. This was especially true for cholestatic liver failure or secondary sclerosing cholangitis [35]. Within our cohort, no imaging features suggestive of sclerosing cholangitis were observed via ultrasound. Additional imaging specific for sclerosing cholangitis (e.g., MRI and MRCP) has not been regularly performed and may be a potential limitation. In the cohort analyzed for this study, long-term elevation of bilirubin in patients with WD and cirrhosis was distinct, but rather nuanced. Whether bilirubin changes may reflect altered cholestasis or rather global liver function impairment is unclear and should therefore be interpreted with caution. The reported modest alteration in bilirubin levels may, however, indeed reflect a decline in overall cirrhosis-associated impairment of liver excretion than an additionally acquired cholestatic disease. In line, long-term transient elastography data were comparable to their respective values prior to infection in both analyzed subgroups. In three patients, the increase in bilirubin levels resulted in Child–Pugh score progression from stage A to stage B. In four cases, bilirubin levels exceeded the clinically relevant threshold of total bilirubin > 2× ULN. However, no episodes of hepatic decompensation were observed during the study period. Overall, these findings suggest limited clinical consequences despite measurable modest bilirubin alterations. Of note, because key assumptions required for parametric repeated-measures analyses were violated and the sample size was limited, parametric models and multivariable adjustments were not conducted. As a result, the analyses may not fully account for potential confounding factors or temporal effects. 

For other chronic liver diseases such as autoimmune hepatitis (AIH), chronic viral hepatitis B or C and liver transplant recipients, necessity of therapy regimen adjustments has been discussed in detail. Overall, consensus is that dosage or regimen adjustment is not necessary in patients who do not suffer from very severe COVID-19. The stability of WD-specific copper parameters and liver stiffness across all time points likely reduced disease-related background noise and made the modest but persistent post–SARS-CoV-2 bilirubin elevations discernible as a potential infection-associated signal, rather than a manifestation of uncontrolled WD progression. It therefore does not seem necessary to adjust dosage and/or the chosen therapeutic agent due to infection with SARS-CoV-2 in patients with WD. However, close monitoring of disease- and pandemic-related new-onset and worsening of pre-existing psychiatric comorbidities is paramount. Recently, it was shown that patients with WD and SARS-CoV-2 infections tend to suffer more often from long-COVID symptoms and that psychiatric diseases are more frequent in this group [23]. These findings combined with the tendency of long-term deterioration of liver cirrhosis in WD patients shown in this group identifies these patients as an especially vulnerable group which should be monitored closely to prevent further health impacts. In light of the structural limitations of our real-world cohort – including major attrition, absence of control groups, delayed post-infection assessments, and the small, selected cirrhotic subgroup – our findings should be viewed as an initial exploratory signal that requires confirmation in larger, controlled studies before any firm clinical implications can be drawn.

## Conclusion

In this study, we show that in patients with WD and cirrhosis, a persistent, modest biochemical alteration in bilirubin levels can be observed after acute SARS-CoV-2 infection. Contrarily, in patients who are diagnosed with WD but do not show any signs of cirrhosis, bilirubin levels were not affected during long-term follow-up. Our findings in cirrhotic WD patients suggest that even mild SARS-CoV-2 infections may be associated with sustained alterations in hepatic excretory function. This observation supports the need for prolonged monitoring after COVID-19 in vulnerable populations.

Therefore, these patients – especially when there is a history of accompanying cirrhosis – should be monitored very closely post-infection to mitigate potential negative effects on both course of the disease and mental health, which ultimately might affect therapy adherence. Nevertheless, these observations are hypothesis-generating and should be interpreted cautiously due to the small, selected cirrhotic subgroup, the lack of appropriate control groups, and the delayed timing of post-infection assessments.

## Supplementary Information

Supplementary Material 1.

## Data Availability

The data generated and analyzed during this study are not publicly available due to potentially identifiable patient information. However, they are available from the corresponding author upon reasonable request.

## Abbreviations

24-UCE: 24 h-urine copper excretion
AIH: Autoimmune hepatitis
ALT/GPT: Alanine-aminotransferase
AP: Alkaline phosphatase
AST/GOT: Aspartate-aminotransferase
BMI: Body Mass Index
CAP: Controlled Attenuation Parameter
ChE: Choline esterase
GGT: Gamma-glutamyltransferase
INR: International normalized ratio
LC: Liver cirrhosis
SARS-CoV-2: Severe acute respiratory syndrome coronavirus 2
SD: Standard deviation
TE: Transient elastography
ULN: Upper limit of normal
WBC: White blood cells
WD: Wilson’s disease
