# Supplementary material for: Exploratory longitudinal cohort study of modest bilirubin-driven biochemical liver alterations after SARS-CoV-2 infection in a selected subgroup of patients with Wilson’s disease and liver cirrhosis
Source: BMC Gastroenterol. 2026 Apr 20;26:241. doi: 10.1186/s12876-026-04840-3 (PMC13097757; doi:10.1186/s12876-026-04840-3)
Supplement: Supplementary file 1 — Supplementary Material 1. [file 12876_2026_4840_MOESM1_ESM.docx]

**Title: Exploratory longitudinal cohort study of modest bilirubin-driven biochemical liver alterations after SARS-CoV-2 infection in a selected subgroup of patients with Wilson’s disease and liver cirrhosis**

**Authors:**

Sebastian Köhrer^1^, Maximilian Lukas Brand^1^, Viola Leidner^1^, Holger Zimmer^2^, Andrea Langel^1^, Jessica Langel^1^, Patrick Michl^1^, Isabelle Mohr^1^

**Affiliations:**

^1^Internal Medicine IV, Department of Gastroenterology, University Hospital Heidelberg, Heidelberg, Germany

^2^Internal Medicine I, Department of Endocrinology, Diabetology, Metabolic Diseases and Clinical Chemistry, University Hospital Heidelberg, Heidelberg, Germany

**Corresponding author:** PD Dr. med. Isabelle Mohr, Department of Gastroenterology, University Hospital Heidelberg, INF 410, 69120 Heidelberg, Phone: +49 6221 56 32818, eMail: [isabelle.mohr@med.uni-heidelberg.de](mailto:isabelle.mohr@med.uni-heidelberg.de)

**Supplemental Table 1: Baseline Characteristics of excluded WD patients.**

| **Category** | **Parameter** | **Value** |
| --- | --- | --- |
| Study population | Patients excluded, n  Male, %  Female, % | 121  45.5 %  54.5 % |
| Demographics | Age at study inclusion, years  Age at diagnosis, years | 38 (range 20 to 69; SD 16.4)  18 (range 3 to 45; SD 9.8) |
| Child-Pugh Score | Stage A, n (%)  Stage B, n (%)  Stave C, n (%) | 24 (19.8)  6 (4.1)  1 (0.8) |
| Clinical phenotype of  WD | Primarily hepatic, n (%)  Primarily neurological, n (%)  Mixed phenotype, n (%) | 85 (70.2)  14 (11.6)  22 (18.2) |
| Treatment at study  inclusion | D-penicillamine, n (%)  Trientine, n (%)  Zinc, n (%) | 25 (25.4)  89 (73.5)  7 (5.7) |
| Disease characteristics | Overall treatment duration, years | 23 (range 4 to 52; SD 14.1) |

**Supplemental Table 2: Summary of questionnaire of new onset symptoms after SARS-CoV-2 infection (defined as Long-COVID symptoms).**

| New Symptom onset after SARS-CoV-2 infection | Long-COVID in WD patients **without liver cirrhosis** | | | | | Long-COVID in WD patients **with liver cirrhosis** | | | | |
| --- | --- | --- | --- | --- | --- | --- | --- | --- | --- | --- |
|  | N=14 out of 49 (28.6%) | | | | | N=2 out of 22 (9.1%) | | | | |
|  | n | n | If **yes (n)**: | | |  |  | If **yes (n):** | | |
|  | **no** | **yes** | mild | moderate | severe | **no** | **yes** | mild | moderate | severe |
| **1 Cough** | 47 | **2** | 2 | 0 | 0 | 22 | **0** | 0 | 0 | 0 |
| **2 Fever** | 49 | **0** | 0 | 0 | 0 | 22 | **0** | 0 | 0 | 0 |
| **3 Shortness of breath**   1. **at rest** 2. **mild activity** 3. **extended acitivity** | 44 | **5** | 1 | 2 | 2 | 20 | **2** | 1 | 1 | 0 |
|  |  | **1** | 1 | 0 | 0 |  | **0** | 1 | 0 | 0 |
|  |  | **2** | 0 | 2 | 0 |  | **0** | 0 | 1 | 0 |
|  |  | **2** | 0 | 0 | 2 |  | **2** | 0 | 0 |  |
| **4 Palpitation** | 45 | **4** | 3 | 0 | 1 | 22 | **0** | 0 | 0 | 0 |
| **5 Chest pain**   1. **at rest** 2. **extended acitivity** | 49 | **0** | 0 | 0 | 0 | 22 | **0** | 0 | 0 | 0 |
|  |  | **0** | 0 | 0 | 0 |  | **0** | 0 | 0 | 0 |
|  |  | **0** | 0 | 0 | 0 |  | **0** | 0 | 0 | 0 |
| **6 Fatigue** | 36 | **13** | 10 | 3 | 0 | 20 | **2** | 0 | 2 | 0 |
| **7 Myalgia** | 47 | **2** | 2 | 1 | 1 | 21 | **2** | 1 | 0 | 0 |
| **8 Athralgia** | 46 | **3** | 1 | 1 | 1 | 20 | **2** | 1 | 1 | 0 |
| **9 Muscular weakness** | 43 | **6** | 4 | 1 | 1 | 20 | **2** | 1 | 1 | 0 |
| **10 Cephalgia** | 43 | **6** | 4 | 2 | 0 | 21 | **1** | 1 | 0 | 0 |
| **11 Vertigo** | 43 | **6** | 4 | 2 | 0 | 22 | **0** | 0 | 0 | 0 |
| **12 Feeling of brain fog** | 42 | **7** | 3 | 3 | 1 | 20 | **2** | 1 | 1 | 0 |
| **13 Anxiety** | 41 | **8** | 5 | 3 | 0 | 21 | **1** | 1 | 0 | 0 |
| **14 Depression** | 40 | **9** | 7 | 1 | 1 | 21 | **1** | 1 | 0 | 0 |
| **15 Sleep disturbances** | 33 | **16** | 13 | 3 | 0 | 20 | **2** | 1 | 1 | 0 |
| **a) falling asleeep**  **b) sleeping through** |  | **14** | 10 | 4 | 0 |  | **0** | 1 | 0 | 0 |
|  |  | **8** | 7 | 1 | 0 |  | **0** | 0 | 1 | 0 |
| **16 Stool disturbances** | 45 | **4** | 2 | 2 | 0 | 22 | **0** | 0 | 0 | 0 |
| 1. **diarrhea** |  | **1** | 1 | 0 | 0 |  | **0** | 0 | 0 | 0 |
| 1. **more obstipation** |  | **3** | 1 | 2 | 0 |  | **0** | 0 | 0 | 0 |
| **17 Dysgeusia** | 49 | **0** | 0 | 0 | 0 | 22 | **0** | 0 | 0 | 0 |
| **18 Dysosmia** | 48 | **1** | 1 | 0 | 0 | 22 | **0** | 0 | 0 | 0 |
| **19 Alopecia** | 44 | **5** | 3 | 1 | 1 | 22 | **0** | 0 | 0 | 0 |
| **20 Sore throat** | 47 | **2** | 2 | 0 | 0 | 22 | **0** | 0 | 0 | 0 |
| **21 Rhinitis** | 45 | **4** | 3 | 1 | 0 | 22 | **0** | 0 | 0 | 0 |
| **22 Exercise capacity** | 41 | **8** | 3 | 4 | 1 | 20 | **2** | 0 | 2 | 0 |
| **23 Blood pressure variations** | 46 | **3** | 2 | 1 | 0 | 22 | **0** | 0 | 0 | 0 |
| **24 Concentration disorders** | 37 | **12** | 9 | 3 | 0 | 20 | **2** | 1 | 1 | 0 |
| **25 Amnestic dysphasia** | 36 | **13** | 10 | 3 | 0 | 21 | **0** | 1 | 0 | 0 |
| **26 Memory disturbances** | 41 | **8** | 7 | 1 | 0 | 20 | **2** | 1 | 1 | 0 |
